# The "New Journal" dilemma

**DOI:** 10.1186/1617-9625-3-2-1

**Published:** 2006-08-15

**Authors:** J Elliott Scott

**Affiliations:** 1 Departments of Oral Biology & Anatomy, University of Manitoba (Winnipeg, Manitoba, Canada)

## 

As we put together another issue of Tobacco Induced Diseases we are faced with a number of publication problems. Within this issue we have several very interesting studies as well as commentaries and reviews all related to smoking or the effects of tobacco smoke components on different biological systems. We begin with a review of a recent publication from the Royal College of Physicians, London that we received and provides an excellent background on passive smoking and the case for clean air. This report is somewhat unique in that it brings together a wide array of information specifically related to environmental tobacco smoke (ETS). It also deals with the economics of smoke-free policies. Probably the most interesting chapters relate to the methods the tobacco industry has used to attempt to negate smoking regulation and again shows that this industry has know of the harmful effects of smoke exposure, both direct and indirect, for many years. The full report is available from the Royal College of Physicians of London (2005).

We next move to a review article by Dr. Bergen concerning the ability of exposure to tobacco smoke or its products during the fetal period to alter maternal energy balance inducing cardiovascular or other diseases in later life. As a researcher involved with regulation of obesity at the neural level through such pathways as leptin activation, Dr. Bergen brings a unique perspective to the question of smoking during pregnancy and provides an interesting bridge between direct smoke exposure levels such as occurs in the daily smoker, characterized by the mother, and the "passive" smoking of the fetus, highlighting the potential of tobacco smoke to induce many, often subtle long term effects.

The presentation of the core of our articles in this issue brings us to the point of this editorial. These articles have undergone extensive peer review and represent the best of scientific publications. Author revisions and further input from reviewers have produced excellent quality scientifically sound pieces of research. But what of other manuscripts we have received that might not yet be considered, for a variety of reasons, to be at a level for publication? The dilemma of the new journal is this: how do we acquired sufficient numbers of publications to produce regular issues and concurrently keep our quality high? What level of scientific publication is acceptable to a new journal and do we have to compromise our standards to achieve full volume publications? We believe the answer to the latter question is clearly no. High quality publications of scientifically sound documentation is a requirement to carve out a niche for an ongoing and credible place for the journal in the scientific world. Indeed those authors who have submitted materials for possible publication which we have been unable to accept should not feel the least slighted. As a new journal we make every effort to develop a support base for these authors and their works. Many publications can eventually reach a level sufficiently high to merit publication and we hope to be able to provide the network of reviewers and authorities that are able and willing to work with these researchers. In addition, as we begin to receive more submissions from authors for whom English is not their first language, we will try to review publications on the basis of scientific merit, the intention being that the language may be improved if the science is sound.

Tobacco Induced Diseases continues on its move to BioMed Central. We appreciate the support of submitting authors.

## Competing interests

The authors declare that they have no competing interests.

